# Expanding use of the Foley catheter in maxillofacial surgery

**DOI:** 10.1308/rcsann.2025.0099

**Published:** 2025-10-07

**Authors:** Z Bhatti, J Thacker

**Affiliations:** ^1^ All India Institute of Medical Sciences, Bhopal, India; ^2^ Karnavati School of Dentistry, Gandhinagar, India

We read with great interest the review by Karmarkar *et al*.^1^ The breadth of surgical applications presented is indeed impressive and we wish to highlight few more applications of the Foley catheter (FC) in oral and maxillofacial surgery.

The FC offers a simple, minimally invasive and effective method for stabilising isolated zygomatic arch fractures following indirect reduction. Zygomatic arch fractures, owing to their prominent facial position, are prone to displacement even after successful reduction, particularly in multi-fragmented cases or because of the activity of the masseter muscle during mouth opening. Conventionally, the Gillies temporal (extraoral) or Keen’s (intraoral) approach achieves reduction using instruments such as Rowe’s or Bristow’s elevator; however, these techniques provide no postoperative stabilisation, predisposing to relapse. To address this limitation, following fracture reduction, an FC is introduced through the same incision, and its balloon is positioned beneath the zygomatic arch. Once inflated, the FC keeps the reduced fragments in alignment and acts as an internal splint. The catheter is generally retained for about 1 week and removed after deflation. Clinical reports by Kumar *et al*^2^ and Sormani Bento Fernandes de Queiroz *et al*^3^ have demonstrated the efficacy of this technique in achieving stable reduction, minimising postoperative displacement, and avoiding additional fixation or scarring – making it a valuable adjunct in zygomatic arch fracture management.

The FC can also be effectively utilised for immobilisation in orbital floor (inferior orbital wall) fractures, especially when the anterior wall of the maxillary sinus is involved. Following reduction of the orbital floor, the catheter balloon can be inserted into the maxillary sinus via a meatal antrostomy or the anterior wall defect, and then inflated to support the reconstructed floor and maintain its contour. The catheter is usually left *in situ* for about 2 weeks, providing gentle internal stabilisation during healing. Park *et al*^4^ reported favourable outcomes using this technique in orbital blowout fractures, demonstrating effective stabilisation, early recovery and minimal complications. Other studies have similarly validated its role as a safe, simple, and inexpensive internal splint in orbital floor reconstruction.

A third innovative use of FC has recently been described in maxillofacial trauma requiring elastic traction or non-rigid maxillomandibular fixation. In many trauma and emergency setups, conventional orthodontic elastics may not be readily available or may lack the tensile strength required for old or complex fractures like condylar fractures. In such cases, customised elastic bands fabricated from the central portion of an FC can serve as a reliable and economical alternative. Kumar *et al*^5^ described preparing 1-mm-wide bands from a 20-Fr FC, which provided effective elastic traction in mandibular fracture management. These elastics are easy to fabricate, adaptable to individual case requirements, and readily available in any hospital setting.

These techniques using FC are reproducible and resource-efficient, especially in settings where specialised materials and instruments are not readily accessible. Thus, we commend the authors for their excellent review and believe that adding these maxillofacial applications will enhance understanding of the FC’s wide surgical usefulness.

## Competing interests

The authors declare no conflict of interest.

## Funding

The authors received no financial support for the research, authorship, and/or publication of this article.

## Author contributions

**Z Bhatti:** Writing – original draft, Writing – review & editing. **J Thacker:** Writing – original draft, Writing – review & editing.

## Artificial Intelligence

The author/s declare that no AI was used to conduct the study or prepare the manuscript.
